# CCL11 promotes migration and proliferation of mouse neural progenitor cells

**DOI:** 10.1186/s13287-017-0474-9

**Published:** 2017-02-07

**Authors:** Feifei Wang, Nobuyasu Baba, Yuan Shen, Tatsuyuki Yamashita, Emi Tsuru, Masayuki Tsuda, Nagamasa Maeda, Yusuke Sagara

**Affiliations:** 1 Center for Innovative and Translational Medicine, Kochi University Medical School, Kohasu, Oko-cho, Nankoku, Kochi 783-8505 Japan; 2 Institute for Laboratory Animal Research, Science Research Center, Kochi University Medical School, Kochi, Japan; 3 Department of Obstetrics and Gynecology, Kochi University Medical School, Kochi, Japan

**Keywords:** Neurogenesis, Neural progenitor cell, Hypoxic-ischemic encephalopathy, CC chemokine

## Abstract

**Background:**

Neonatal hypoxia-ischemia induces massive brain damage during the perinatal period, resulting in long-term consequences to central nervous system structural and functional maturation. Although neural progenitor cells (NPCs) migrate through the parenchyma and home in to injury sites in the rodent brain, the molecular mechanisms are unknown. We examined the role of chemokines in mediating NPC migration after neonatal hypoxic-ischemic brain injury.

**Methods:**

Nine-day-old mice were exposed to a 120-minute hypoxia following unilateral carotid occlusion. Chemokine levels were quantified in mouse brain extract. Migration and proliferation assays were performed using embryonic and infant mouse NPCs.

**Results:**

The neonatal hypoxic-ischemic brain injury resulted in an ipsilateral lesion, which was extended to the cortical and striatal areas. NPCs migrated toward an injured area, where a marked increase of CC chemokines was detected. In vitro studies showed that incubation of NPCs with recombinant mouse CCL11 promoted migration and proliferation. These effects were partly inhibited by a CCR3 antagonist, SB297006.

**Conclusions:**

Our data implicate an important effect of CCL11 for mouse NPCs. The effective activation of NPCs may offer a promising strategy for neuroregeneration in neonatal hypoxic-ischemic brain injury.

**Electronic supplementary material:**

The online version of this article (doi:10.1186/s13287-017-0474-9) contains supplementary material, which is available to authorized users.

## Background

Perinatal hypoxia and ischemia can cause serious complications. In neonatal animal models, accumulating evidence suggests that hypoxic-ischemic brain damage influences the activation of endogenous neural progenitor cells (NPCs) in the subventricular zone (SVZ) [1]. Increased neurogenesis and migration of NPCs toward the injury site has been observed in animal models of epilepsy, stroke, trauma, Alzheimer’s disease, Parkinson’s disease, and Huntington’s disease [2]. Recent studies indicate that neurogenesis may play regenerative roles in response to central nervous system (CNS) injuries. Neurogenesis in the immature mammalian brain is highly modulated by excitotoxic insults [3, 4]. Moreover, NPCs have been shown to be induced in the post-stroke human cerebral cortex for more than 1 month based on immunostaining of autopsied tissue from stroke patients [5, 6]. Although the origin of these NPCs is uncertain, this raises the possibility of developing therapeutic strategies aimed at activating the neurogenic capacity in order to repair the damaged brain.

Nevertheless, the molecular mechanisms for NPC activation and mobilization have not been clearly identified. It has been demonstrated in rodents that repulsive Slit-Robo signal orientated the migration of newly generated immature neurons through the rostral migratory stream (RMS) under normal conditions. RMS astrocytes express Robo receptors and regulate the rapid migration of Slit1-expressing new neurons [7]. Fibroblast growth factor 2 (FGF-2) and anosmin-1 increase SVZ NPC migration via epidermal growth factor receptor 1 (EGFR1) during pre- and postnatal rat development [8]. In contrast, in the injured brain, NPCs from the SVZ migrate to injury sites through blood vessels for up to 1 year after injury [9, 10]. This directed NPC migration is considered the result of chemoattractive cues expressed from the injury site. Inflammatory mediators contribute to the pathological changes, which are sensitive to hypoxic-ischemic brain injury [11].

We hypothesized that inflammatory mediators were involved in the migration of NPCs toward the injury site. The major role of chemokines is to act as a chemoattractant to guide the migration of immune cells. The CC chemokines induce recruitment of eosinophils, basophils, neutrophils, and monocytes, and play important roles in the regulation of many inflammatory conditions. In this study, we demonstrated that CCL11, one of CC chemokines highly expressed at brain injury sites, promoted the migration and proliferation of NPCs. These findings suggest that CCL11 may have important functions in the neonatal CNS.

## Methods

### In vivo study

#### Neonatal ischemia-reperfusion brain injury

The NOD/SCID (NOD.CB17-Prkdc^scid^/J) mice were purchased from Charles River Laboratories Japan Inc. (Kanagawa, Japan). Experimental mouse models of neonatal ischemia-reperfusion brain injury were used based on a modified Rice-Vannucci model as previously described [12]. Briefly, 9-day-old postnatal NOD/SCID mice of both sexes (n = 48) were anesthetized with 2% isoflurane. The right common carotid artery (CCA) was occluded with an aneurysm clip (Mizuho, Tokyo, Japan). The pups were placed in a hypoxia chamber held at 8% O_2_ for 120 minutes. Reperfusion was achieved by unclamping the artery and exposing the pups to normoxic conditions. Pups were returned to their dams after the procedure. Sham-operated mice (n = 8) were subjected to CCA isolation alone and no brain injury. NOD/SCID mice aged 1, 2, 4, 6, and 8 weeks were used as normal control (n = 30). All animal experiments were performed under an institutionally approved protocol for the use of animal research at Kochi University (approval number: J-00002).

#### Magnetic resonance imaging

Magnetic resonance imaging (MRI) measurements were performed on a Varian 9.4 T vertical MR system with console Vnmr interface (Agilent Technologies, Santa Clara, CA, USA). The mice were anesthetized with 2% isoflurane and placed in a vertical animal positioning unit. The MRI sequence used was a T2-weighted Spin Echo Multi Slice (SEMS) imaging sequence (T2WI) with the following parameters: repetition time (TR), 2000 ms; echo time (TE), 10 ms; 1 mm thick slices; number of scans, 9; field of view (FOV), 2 × 2 cm; and matrix size, 256 × 128. The total imaging time for each sequence was 16 minutes.

#### Immunohistochemistry

At each follow-up time point, the brains were removed and fixed in 4% paraformaldehyde. Coronal brain sections (12 μm) were prepared using a microtome (Leica Microsystems Inc., Wetzlar, Germany). The primary and secondary antibodies used in the experiments were: rabbit anti-Doublecortin (Dcx) antibody (1:1000, Abcam, Cambridge, UK), mouse anti-polysialylated neural cell adhesion molecule (PSA-NCAM) antibody (1:200, Merck Millipore, Darmstadt, Germany) and Alexa Fluor® 488 and 594 goat anti-rabbit IgG (1:1000, Thermo Fisher Scientific Waltham, MA, USA). Slides were mounted with ProLong™ Diamond Anti-fade Mountant with DAPI (Life Technologies, Carlsbad, CA, USA). The slides were observed using a fluorescence microscope (BZ-9000; Keyence, Osaka, Japan). Dcx and PSA-NCAM-positive cells were counted per slice.

#### Chemokine quantification

The brain tissue of mice were collected at each follow-up time point. The lysate was prepared by homogenization in RIPA Lysis and Extraction Buffer (Thermo Fisher Scientific) containing a complete protease inhibitor cocktail (Roche, Basel, Switzerland). The tissue debris was removed by centrifugation at 1000 × g for 10 minutes at 4 °C. Protein concentration was determined with BCA Protein Assay Kit (Thermo Fisher Scientific).

Chemokine levels in RIPA-extracted lysates were quantified using CCL9 ELISA Kit (Sigma-Aldrich, St. Louis, MO, USA), CCL12 Quantikine ELISA Kit (R&D Systems, Minneapolis, MN, USA), and LEGENDplex Multi-Analyte Flow Assay Kit for Mouse Proinflammatory Chemokine (BioLegend, San Diego, CA, USA) according to the manufacturer’s instructions. Data were normalized to the expression level in the 1 mg/ml of total protein of the lysates. The relative detection threshold of those assays was 0.2 pg/ml except the CCL12 assay, which was 1.5 pg/ml.

#### Neutralizing assay

A neutralizing rat monoclonal CCL11 antibody (R&D Systems) at a dose of 25 μg/mouse was administrated twice at 6 and 24 hours after brain injury. At 1 week after injury, the brains were removed and fixed in 4% paraformaldehyde. The primary and secondary antibodies used in the experiments were: rabbit anti-Dcx antibody (1:1000, Abcam) and Alexa Fluor™ 488 goat anti-rabbit IgG (1:1000, Thermo Fisher Scientific). The slides were observed using a fluorescence microscope (BZ-9000; Keyence).

### In vitro study

#### Isolation and culture neural stem/progenitor cells

Embryonic NPCs were isolated from pregnant NOD/SCID mice (n = 3) on E13.5 day of gestation. Striata were dissected from each embryo in phosphate-buffered saline (PBS) containing 0.6% glucose (Sigma-Aldrich) and penicillin-streptomycin (50 U/ml; Life Technologies). After dissection, the tissue was triturated to a single cell suspension using a transfer pipette. Cells were cultured in NeuroCult™ Proliferation Medium containing 20 ng/ml epidermal growth factor (EGF) (Stemcell Technologies Inc., Vancouver, BC, Canada). NPCs from infant NOD/SCID mice were isolated from the ipsilateral and contralateral SVZ at 1 week post-injury (n = 5). The SVZ tissue was triturated with neural tissue dissociation solution (Sumitomo Dainippon Pharma Co., Ltd, Tokyo, Japan). Cells were cultured in NeuroCult™ Proliferation Medium containing 20 ng/ml EGF, 10 ng/ml fibroblast growth factor (FGF) and 2 μl/ml heparin (Stemcell Technologies Inc.). Cells were proliferated to generate neurospheres.

#### RT-PCR

Total RNA was isolated from neurospheres using an RNeasy Mini kit (Qiagen, Hilden, Germany) according to the manufacturer’s instruction. RT-PCR was performed using PrimeScript RT reagent kit (Takara Bio Inc., Otsu, Japan) for reverse transcription, and Ex Taq (Takara Bio Inc.) for PCR amplification. Details of primer sequences are shown in Table 1.

**Table 1:** Primer sequence for RT-PCR

Gene	Primer sequences
SOX2	F: 5′-CTGGCAAGACCGTTTTCGTG-3′
	R: 5′-ATTCTCGGCAGCCTGATTCC-3′
Ki67	F: 5′-AAGCAAACCAGCTGCAGAAA-3′
	R: 5′-TTGGATAGGACAGAGGGCCA-3′
Dcx	F: 5′-CGGAAGCACAAGGACCTGTA-3′
	R: 5′-AAGGCCCCTAAGCATTCAGT-3′
NeuN	F: 5′-CCTCCGGGAAAATTGGCTGA-3′
	R: 5′-TTATTGACCTTGGAGCCCCG-3′
GFAP	F: 5′-ATGCGGGATGGTGAGGTCAT-3′
	R: 5′-GCCTCAGGGACTTTCCCTTT-3′
CCR3	F: 5′-ATGGCATTCAACACAGATG-3′
	R: 5′-AATCCAGAATGGGACAGTG-3′
ACTB	F: 5′-AGATCAAGATCATTGCTCCTCCT-3′
	R: 5′-TTTGGGGGATGTTTGCTCCA-3′

#### Flow cytometry

Expression of NPC surface markers was analyzed using the LSR Fortessa (Becton Dickinson, San Jose, CA, USA). NPCs were plated onto poly-D-lysine coated T-25 culture flasks. At 24 hours after seeding, NPCs were collected and washed twice with staining buffer, and resuspended in 100 μL of PE-conjugated anti-mouse PSA-NCAM antibody (Miltenyi Biotec Inc., Bergisch Gladbach, Germany, 1:11) or PE-conjugated mouse IgM isotype control antibody (Miltenyi Biotec Inc., 1:50). Data was evaluated using FlowJo software (Tree Star, Inc., Ashland, OR, USA).

#### Migration assay

Carrier-free forms of mouse recombinant CCL2, CCL3, CCL4, CCL5, CCL9, CCL11, CCL12, CCL20, and CCL22 were purchased from BioLegend (San Diego, CA, USA). Assays utilized a poly-D-lysine coating μ-slide Chemotaxis^3D^ (ibidi GmbH, Munich, Germany). 3 × 10^6^ cells/ml were seeded onto the μ-slides and incubated at 37 °C. Migration was stimulated by addition of the chemokine (20 μg/ml) dissolved in PBS to the indicated port. Time-lapse imaging was performed using BioStation IM-Q (Nikon, Tokyo, Japan) with a × 20 objective. Images were captured every 15 minutes for 24 hours. Twelve cells were randomly chosen in each chemokine assay for data analysis. The migration trajectory and distance were measured with ImageJ using the Manual Tracking plugin for NIH ImageJ software (Bethesda, MD, USA).

**Fig. 1:**
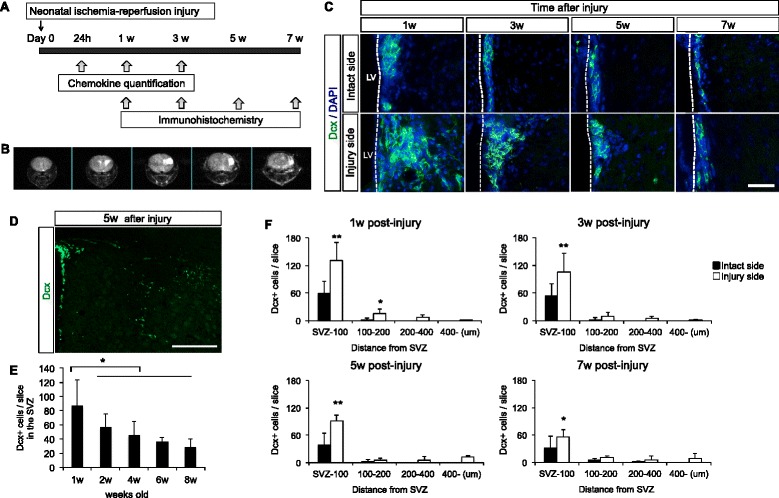
The proliferation and migration of Dcx-positive cells in a mouse model of neonatal ischemia-reperfusion brain injury. **a** The timeline of the study design. **b** Serial coronal T2-weighted MR images at 1 week after brain injury. T2 hyperintensity abnormalities were observed in the cortex and striatum. Five coronal slices were acquired with 1 mm thickness. **c** Fluorescent image of the ipsilateral (injury) and contralateral (intact) side of SVZ in the model mouse. In the injured SVZ side, the number of Dcx-positive cells began to increase rapidly at 1 week, and was stable thereafter for up to 7 weeks following the injury. *Green*: Dcx; *blue*: DAPI. Scale bar = 50 μm. *LV* lateral ventricle. **d** Dcx-positive cells were migrated to the injured site at 5 weeks post-injury. *Green*: Dcx. Scale bar = 200 μm. **e** In the intact SVZ side, the number of Dcx-positive cells was diminished with advancing age. N = 5 for each time point. The data are presented as the mean numbers of positive cells in the SVZ ± S.D. ^*^ *P* < 0.05. **f** Number of Dcx-positive cells per slice in 1–7 weeks post-injury. Abundant Dcx-positive cells were distributed from the SVZ laterally toward the damaged area. N = 6 for each time point. The data are presented as the mean numbers of positive cells in the ischemic hemisphere (injury side), and the contralateral hemisphere (intact side) ± S.D. ^*^ *P* < 0.05, ^**^ *P* < 0.01

#### Cell culture assay

Embryonic and infant NPCs were plated onto 96-well plates at a cell density of 1 × 10^4^ cells/well. Each chemokine (2 μg/ml) was added to cell cultures. The culture medium used was NeuroCult™ Proliferation Medium without EGF, FGF, and heparin. After 1 and 3 days of culture, 10 μl of CCK-8 (Dojindo, Kumamoto, Japan) solution was added in triplicate to wells, and incubated for 2 hours at 37 °C. Absorbance at 450 nm was then measured in a microplate reader (Thermo Fisher Scientific).

#### Immunocytochemistry

NPCs were fixed with 4% paraformaldehyde before migration assay. After fixation, mouse anti-Dcx antibody (1:250) and rabbit anti-CCR3 antibody (1:250, Abcam) were applied at 4 °C overnight. Alexa Fluor™ 488-conjugated anti-mouse IgG (1:500) and Alexa Fluor™ 594-conjugated anti-rabbit IgG (1:500, Thermo Fisher Scientific) were used as the respective secondary antibody. The cells were observed using a fluorescence microscope (BZ-9000; Keyence).

#### CCR3 blocking assay

To evaluate CCR3 blocking, NPCs were incubated with the CCR3 antagonist SB 297006 (100 μM) for 30 minutes and then stimulated with CCL11 (2 μg/ml). After 3 days of culture, NPCs were subjected to CCK-8 assays.

#### Statistical analysis

The data are presented as means ± S.D. Results from different groups were compared using the Student’s *t* test, or one-way ANOVA followed by Dunnett’s multiple comparison tests. *P* < 0.05 was considered to indicate a statistically significant difference.

## Results

### NPCs migration in the mouse brain

The experimental design is showed in Fig. 1a. The mouse model of neonatal ischemia-reperfusion injury was induced at post-natal day 9. MRI was performed 1 week after the brain injury. T2 hyperintensity abnormalities reflecting tissue edema were observed in the cortex and striatum of the mouse brain (Fig. 1b). We evaluated the migration of NPCs (Dcx-positive cells) in the neonatal mice brain (Fig. 1c). In the injured SVZ side, the number of Dcx-positive cells began to increase rapidly at 1 week, and was stable thereafter for up to 7 weeks following the brain injury (Fig. 1c, f). At 5 weeks post-injury, Dcx-positive cells had migrated a long distance and had reached the core injury area (Fig. 1d). In contrast, in the intact SVZ side, the number of Dcx-positive cells was diminished with advancing age (Fig. 1e). The number of PSA-NCAM-positive cells was also significantly increased in the injured side (Additional file 2: Figure S2a, b). In the sham-operated mice, there was no difference in the number of Dcx-positive cells between the intact side and the injury side (Additional file 2: Figure S2c, d).

**Fig. 2:**
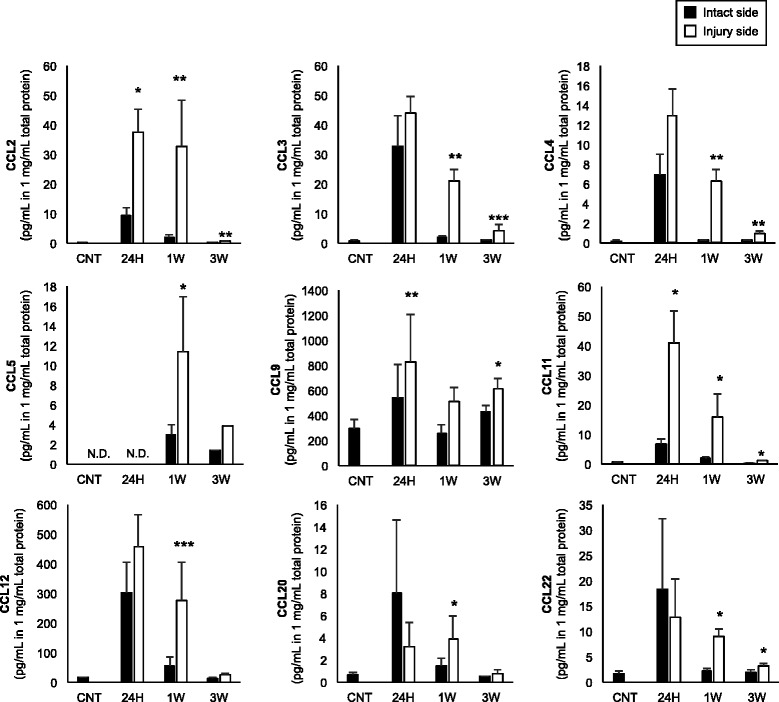
Chemokine quantification. Nine CC chemokines were quantified in the brain tissue extract. In the injury side, most chemokines were significantly upregulated at 24 hours post-injury compared with the intact side. Samples prepared from healthy (non-manipulated) mice were used as control (CNT). N = 4 for each time point. The data are presented as the mean of chemokine secretion in the ischemic hemisphere (injury side), and the contralateral hemisphere (intact side) ± S.E.M. ^*^ *P* < 0.05, ^**^ *P* < 0.01, ^***^ *P* < 0.005. *N.D.* not detected

### Chemokine expression in the mouse brain

Chemokine levels were quantified using ELISA Kit and Multi-Analyte Flow Assay Kit for Mouse Proinflammatory Chemokine. We focused on the expression profile of nine CC chemokines upregulated in the injured mouse brain: CCL2, CCL3, CCL4, CCL5, CCL9, CCL11, CCL12, CCL20, and CCL22. At 24 hours post-injury, most chemokines were highly expressed even in the intact side of the injured brain compared with the undamaged brain (CNT). At 1 week post-injury, CCL2, CCL3, CCL4, CCL5, CCL11, CCL12, CCL20, and CCL22 were significantly upregulated in the injury side, although there was little difference between the injury and intact side in most chemokines at 3 weeks post-injury. Only CCL9 expression was maintained at high levels in both sides of brain up to 3 weeks post-injury (Fig. 2).

**Fig. 3:**
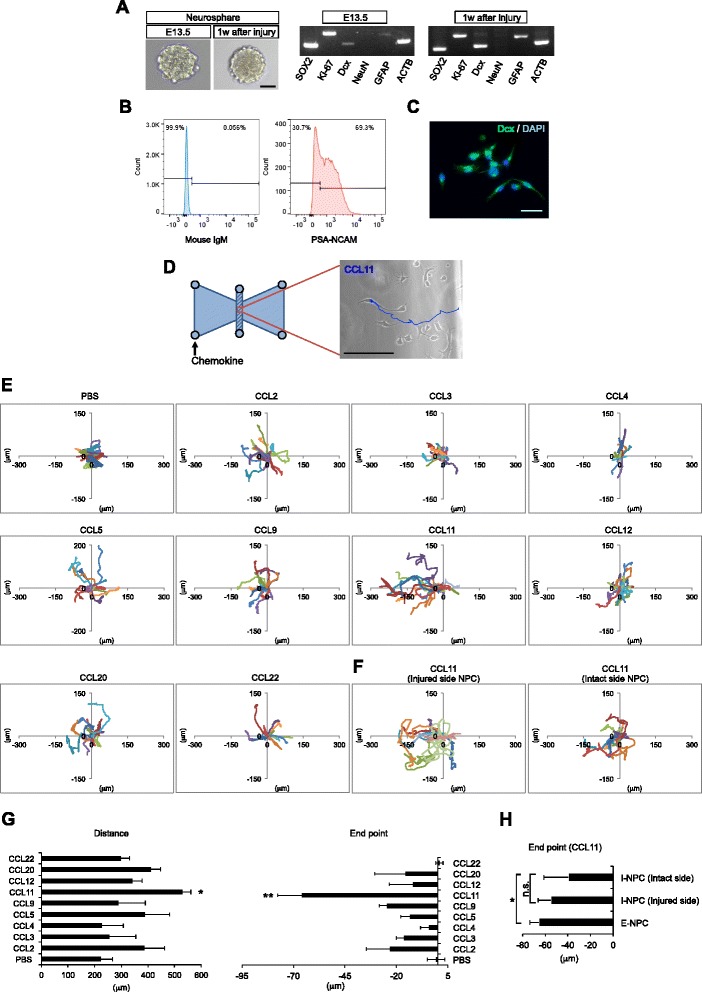
Isolation of NPCs and migration assay. **a** The neurosphere from embryonic and infant mouse brains. Genes involved in proliferation and multipotency were expressed in cultured NPCs. Scale bar = 50 μm. **b** Flow-cytometric analysis for NPCs. The *left panel* showed an isotype control mouse IgM antibody. The *right panel* showed that PSA-NCAM was expressed in NPCs. **c** Immunostaining of Dcx for embryonic NPCs. Dcx-positive cells showed spindle cell morphology. *Green*: Dcx; *blue*: DAPI. Scale bar = 30 μm. **d** Migration assay using μ-Slide Chemotaxis. NPCs were seeded at 3 × 10^6^ cells/ml in the shaded area. Chemokine was injected through the indicated port. Time-lapse imaging and tracking of NPCs with CCL11 (*blue line*). Scale bar = 100 μm. **e**, **f** The trajectory of randomly selected embryonic (**e**) and infant (**f**) NPCs in migration assay. Each color represents the trajectory of an individual cell. The y-axis and the x-axis represents migration distance (μm). The minus (leftward) direction of cell movement is defined as chemokine-induced migration. **g** The distance and end point of embryonic NPCs migration. CCL11 significantly increased the migration distance of NPCs compared with the control (PBS) and other chemokines. The data are presented as the mean of end points ± S.D. The migration assay was repeated three independent times per chemokine. ^*^ *P* < 0.05, ^**^ *P* < 0.01. **h** The end point of trajectory of the embryonic NPCs (*E-NPC*) and infant NPCs (*I-NPC*) derived from the injury and intact side of mouse brain using CCL11. ^*^ *P* < 0.05. *n.s.* not significant

**Fig. 4:**
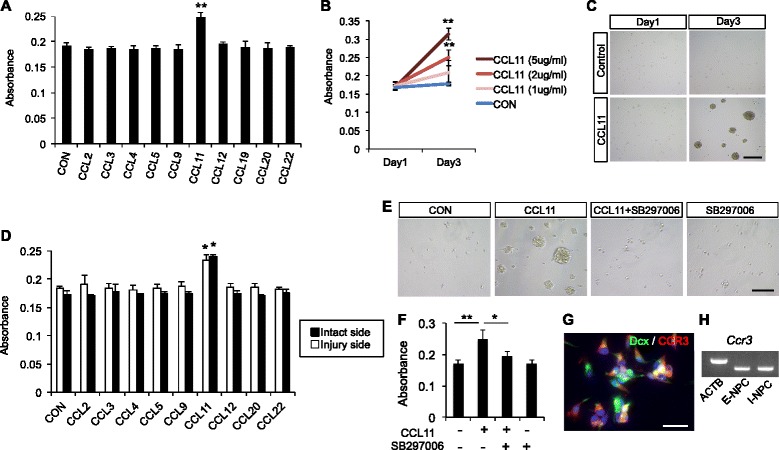
The cell culture assay for NPCs. **a** Proliferation of embryonic NPCs was enhanced after incubation with 2 μg/ml CCL11 at 3 days post-treatment. Other CC chemokines neither formed the neurospheres nor enhanced NSC proliferation. The data are presented as the mean absorbance of three replicates ± S.D. ^**^ *P* < 0.01. **b** The concentration-dependent effect of CCL11 on NPC proliferation. High concentration of CCL11 (2 μg/ml, 5 μg/ml) promoted NPC proliferation. ^**^ *P* < 0.01 vs control (PBS). **c** NPCs treated with 2 μg/ml CCL11 formed neurospheres at 3 days post-treatment. Scale bar = 50 μm. **d** CCL11 (2 μg/ml) enhanced proliferation of infant NPCs derived from both injury and intact sides of the mouse brain. The data are presented as the mean absorbance of three replicates ± S.D. ^*^ *P* < 0.05. **e**, **f** The CCR3 receptor antagonist SB 297006 blocked the CCL11-induced NPC proliferation and neurosphere formation. Scale bar = 50 μm. ^*^ *P* < 0.05, ^**^ *P* < 0.01. **g** Merged image of double immunostaining of Dcx and CCR3 for embryonic NPCs. *Green*: Dcx; *red*: CCR3, *blue*: DAPI. Scale bar = 30 μm. **h** RT-PCR analysis of CCR3 expression in NPCs. CCR3 are expressed in embryonic and infant NPCs

### In vitro migration assay

Neurospheres were generated from the embryo and infant mice brains, and these showed high expression of sex-determining region Y-box 2 (SOX2), Ki-67, and no expression of the mature neuronal marker, NeuN (Fig. 3a). NPCs cultured on poly-D-lysine expressed the neuroblast maker, PSA-NCAM (PSA-NCAM^+^ cells: 68.7 ± 5.2%, PSA-NCAM^-^ cells: 30.2 ± 2.9%) and Dcx (Fig. 3b, c). We then evaluated the effect of chemokines on NPC migration. Cell tracking with CCL11 over 24 hours of monitoring is shown in Fig. 3d. The minus (leftward) direction of cell movement was defined as chemokine-induced migration (Fig. 3e). Each color represents the trajectory of an individual cell. NPCs actively proliferated and migrated toward the CCL11 injected side (Fig. 3e, Additional file 1: Figure S1). When migration distance and end points of embryonic NPCs between each chemokine were compared, CCL11 significantly promoted embryonic NPCs migration compared with PBS or other chemokines (Fig. 3g, PBS: -0.77 ± 4.3 μm; CCL11: -65.9 ± 11.6 μm). Although CCL11 also promoted migration of NPCs derived from the brain-injured infant mice, the end points of migration were decreased in the infant NPCs compared to embryonic NPCs. There was no significant difference between the injury side and intact side of NPCs from infant brain (Fig. 3f, h).

### Cell culture assay

Proliferation of embryonic NPCs was enhanced after incubation with recombinant mouse CCL11 (Fig. 4a). Moreover, the proliferative effect of CCL11 was concentration-dependent. Only NPCs incubated with CCL11 formed neurospheres, and other CC chemokines neither induced neurosphere formation nor enhanced NPC proliferation (Fig. 4b, c). CCL11 also enhanced the proliferation of infant NPCs. There was no significant difference in proliferation activity between NPCs derived from the injury side and those from the intact side of the infant mouse brain (Fig. 4d).

**Fig. 5:**
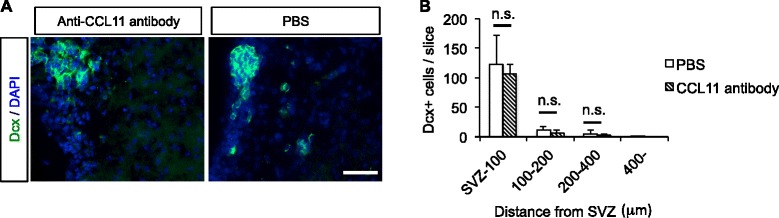
The neutralizing assay in the model mice of neonatal hypoxic-ischemic brain injury. **a** Fluorescent image of the ipsilateral (injury) side of SVZ in model mouse. *Green*: Dcx; *blue*: DAPI. Scale bar = 100 μm. **b** Number of Dcx-positive cells per slice in the model mice. N = 4. The data are presented as the mean numbers of Dcx-positive cells in the ischemic hemisphere (injury side) ± S.D. *n.s.* not significant

### Receptor blocking assay

The major receptor for CCL11 is CCR3, which is found on leukocytes [13]. We then analyzed whether chemokine/receptor signaling influenced the proliferation of NPCs. NPCs expressed a functional chemokine receptor CCR3, and CCR3 receptor antagonist, SB297006 significantly inhibited proliferation and neurosphere formation in CCL11-treated NPCs (Fig. 4e-h).

### In vivo neutralizing assay

A neutralizing CCL11 antibody was administrated in the model mice at 6 and 24 hours after injury. There was no significant difference of NPC migration and proliferation between the antibody-treated group and the PBS-treated group (Fig. 5a, b).

## Discussion

### NPCs migration and chemokines in an injured brain

A number of studies have shown activation and recruitment of NPCs in animal models of ischemic brain injury [14–16]. The number of NPCs correlated with the volume of brain injury in the mouse model of stroke [17]. While low oxygen increased the proliferation of neural stem cells in vitro [18], oxygen glucose deprivation (OGD) decreased the growth of NPCs and also decreased the phosphorylation of extracellular signal regulated kinase (ERK) [19]. The hypoxic-ischemic condition depletes the ATP content of the cells, compromising their metabolic activity. Various inflammatory mediators (cytokines, chemokines, and adhesion molecules) are produced from immune cells, endothelial cells, fibroblasts, and brain cells after ischemic injury. Directed NPC migration is considered to be the result of chemoattractive cues expressed from the injury site. Our chemokine quantification data demonstrated that CC chemokines were markedly elevated at 24 hours after injury, and were gradually reduced over 3 weeks post-injury in the infant mouse brain. Peculiarly, CCL2 and CCL11 were significantly upregulated in the injury side compared with the intact side up to 3 weeks post-injury. Chemokines are 8–14 kDa small molecules mainly regulating immune cells trafficking during inflammatory responses. Chemokine-mediated signaling leads to cytoskeletal rearrangements that allow cell polarization toward the chemokine gradient. In recent years, chemokines have been reported to have non-immunological effects in the CNS, including regulation of cell proliferation, migration, survival, and synaptic activity [20]. Adult NPCs have been shown to express chemokine receptors, including CCR1-8, 10, and CXCR1-6 [21]. Among chemokine receptors, CXCL12/CXCR4 signaling is important for neural progenitor cell guidance and orientation in the developing mammalian brain [22, 23]. Following neural damage, CXCL12/CXCR4 signaling mediates specific migration of NPCs to the ischemic damaged site [24]. Several signaling pathways may mediate CXCL12-induced NPC migration, including inositol 1,4,5-triphosphate, extracellular signal-regulated kinases 1/2, Akt, c-Jun N-terminal kinase, and intracellular calcium [25]. CCL2, CCL3, and CXCL1 also promote NPC migration to the striatum following quinolinic acid-induced lesion [26]. IFN-γ and IFN-β both inhibit cultured adult NPCs proliferation, but only IFN-γ promotes neuronal differentiation [27]. In the present study, CCL2 and CCL3 were upregulated in the injury side in the hypoxic-ischemic brain of neonatal mice. The migration assay showed that CCL2 and CCL3 slightly promoted NPC migration. Since NPCs are placed into the gap of μ − slide Chemotaxis^3D^ and exposed to linear concentration gradients, the concentration of chemokines in the NPC area might be quite diluted. In consideration of a local inflammatory response, the induction effect may vary with the concentration of each chemokine in the injured mouse brain. The results of in vivo neutralizing assay suggests that the activation and migration of NPCs seems to be controlled by an orchestration of cytokines include interleukins, interferons, growth factors and chemokines in the brain-injured mice.

In another study, induction of striatal neurogenesis by the intraventricular administration of brain-derived neurotrophic factor (BDNF) and EGF promoted functional recovery in a mouse model of neonatal hypoxic-ischemic brain injury. In contrast, this BDNF/EGF-associated functional recovery was abolished in mice receiving a co-infusion of 2% cytosine-b-D-arabinofuranoside (Ara-C), a mitotic inhibitor. These results indicate that the effect of functional recovery may be the result of newly generated neurons [28]. Identification of more factors responsible for recruitment of NPCs to the injury site may lead to the development of therapies designed to promote functional recovery after neonatal ischemia-reperfusion brain injury.

### Role of CCL11 in CNS

Whereas chemokine signaling in immune cells has been studied in detail, the molecular mechanisms of chemokine-induced NPC activation are not completely understood. This study has shown that a chemokine, CCL11, could activate NPCs in vitro. The CCL11 activity for NPCs was exerted under EGF- and FGF-free conditions. CCL11 is traditionally associated with eosinophil recruitment and pro-inflammatory responses [29, 30]. The mechanism of how CCL11 affects the brain has not been determined. Recently, increased blood levels of CCL11 in aging have been shown to negatively regulate adult hippocampal neurogenesis. Systemic CCL11 administration decreased the numbers of Dcx-positive cells in the dentate gyrus of young mice. Young mice that received CCL11 exhibited impaired learning and memory deficits [31]. In aged mice, plasma CCL11 protein levels were significantly increased in 18-month-old mice than in 21-month-old mice [32]. In the mice model of experimental cerebral malaria, CCL11 was elevated in the hippocampus and frontal cortex. Dcx-positive cells in the dentate gyrus were decreased in parallel with increased CCL11 [33]. Furthermore, CCL11 promoted the migration of microglia, and induced microglial production of reactive oxygen species. CCL11 triggers oxidative stress via microglial activation and potentiates glutamate-mediated neurotoxicity, which may be involved in the pathogenesis of various neurological disorders [34]. In one physiological study, the striatum had an early rapid-uptake phase for CCL11, which was the fastest among different brain regions [35]. CCL11 in the circulation can cross many regions of the blood-brain barrier (BBB). This suggests that blood-borne CCL11 may have important physiological functions in the CNS and implicates the BBB as an important regulator of the physiological versus pathological effects of this chemokine. Our results may reflect opposite effects of CCL11 on NPCs, as recently reported in previous studies. In our study, the proliferation of NPCs was promoted at a much higher concentration (1–5 μg/ml) of CCL11 compared to Villeda’s study (10 ng/ml) [31]. Moreover, CCL11 might have differential effects in SVZ NPCs compared to hippocampal NPCs.

Meanwhile, positive effects of CCL11 have also been reported in several studies. Adzemovic et al*.* demonstrated that the CCL11/CCR3 pathway was associated with immune response modulation in a rat model of multiple sclerosis (MS). The milder EAE phenotype in the Eae18b congenic strain of rat was accompanied by significantly lower myelin loss and reduced cumulative microglia activation. The congenic rats significantly upregulated CCL11 mRNA in lymph nodes and spinal cord tissue [36]. Moreover, CCL11/CCR3 promotes the proliferation of oligodendrocyte precursor cells (OPCs) and migration of smooth muscle cells (SMCs) [37, 38]. Both of these cells are involved in the regeneration of damaged tissue. In demyelinating diseases such as MS, remyelination is preceded by the division of endogenous NG2-positive OPCs. The OPCs migrate to sites of injury and differentiate into mature oligodendrocytes [39, 40]. In atherosclerosis, SMCs proliferate and migrate to form part of the intimal plaque [41]. Furukawa et al. showed that IL-10, an anti-inflammatory cytokine elevation was correlated with milder symptoms and that CCL11 elevation was correlated with slower disease progression in patients with amyotrophic lateral sclerosis (ALS), suggesting that those cytokines may confer neuroprotection against ALS [42]. Therefore, CCL11 may have both beneficial and harmful effects in the CNS that depend on factors such as concentration, anatomical location, and pathological condition.

## Conclusions

We have investigated the mechanisms for NPC activation in a mouse model of neonatal ischemia-reperfusion brain injury. Our main findings are that CCL11 affects the migration and proliferation of NPCs. Further studies are needed to delineate the regulatory pathways and downstream signaling. The activation of NPCs may offer a promising strategy for neuroregeneration in neonatal hypoxic-ischemic brain injury and general neurological disorders as well.

## Additional files

Additional file 1: Figure S1. The time-lapse movie of NPC migration. Images were captured every 15 minutes for 24 hours. (A) NPCs did not migrate toward the PBS-injected side. (B) After CCL11 injection, NPCs actively proliferated and migrated toward the CCL11-injected side. (ZIP 144651 kb)
Additional file 2: Figure S2. The immunostaining in the model mice of neonatal hypoxic-ischemic brain injury and the sham-operated mice. (A) PSA-NCAM immunostaining image of SVZ in the model mouse. *Red*: PSA-NCAM; *blue*: DAPI. Scale bar = 50 μm. (B) Number of PSA-NCAM-positive cells per slice. N = 8. The data are presented as the mean numbers of PSA-NCAM-positive cells in the injury side and the intact side ± SD. ^*^ *P* < 0.05. (C) Dcx immunostaining image of SVZ in the sham-operated mouse. N = 6. *Green*: Dcx; *blue*: DAPI. Scale bar = 50 μm. (D) Number of Dcx-positive cells per slice. N = 8. The data are presented as the mean numbers of Dcx-positive cells in the sham-operated side and the intact side ± SD. *n.s.* not significant. (PDF 655 kb)

## Abbreviations

ALS: Amyotrophic lateral sclerosis
BBB: Blood-brain barrier
BDNF: Brain-derived neurotrophic factor
CCA: Common carotid artery
CNS: Central nervous system
Dcx: Doublecortin
EGF: Epidermal growth factor
EGFR1: Epidermal growth factor receptor 1
FGF: Fibroblast growth factor
MRI: Magnetic resonance imaging
MS: multiple sclerosis
NPC: Neural progenitor cell
PBS: Phosphate-buffered saline
PSA-NCAM: Polysialylated neuronal cell adhesion molecule
RMS: Rostral migratory stream
SOX2: Sex-determining region Y-box 2
SVZ: Subventricular zone

## References

[1] Kadam SD, Mulholland JD, McDonald JW, Comi AM (2009). Poststroke subgranular and rostral subventricular zone proliferation in a mouse model of neonatal stroke. J Neurosci Res.

[2] Parent JM (2003). Injury-induced neurogenesis in the adult mammalian brain. Neuroscientist.

[3] Plane JM, Liu R, Wang TW, Silverstein FS, Parent JM (2004). Neonatal hypoxic-ischemic injury increases forebrain subventricular zone neurogenesis in the mouse. Neurobiol Dis.

[4] Ong J, Plane JM, Parent JM, Silverstein FS (2005). Hypoxic-ischemic injury stimulates subventricular zone proliferation and neurogenesis in the neonatal rat. Pediatr Res.

[5] Jin K, Wang X, Xie L, Mao XO, Zhu W, Wang Y, Shen J, Mao Y, Banwait S, Greenberg DA (2006). Evidence for stroke-induced neurogenesis in the human brain. Proc Natl Acad Sci U S A.

[6] Nakayama D, Matsuyama T, Ishibashi-Ueda H, Nakagomi T, Kasahara Y, Hirose H, Kikuchi-Taura A, Stern DM, Mori H, Taguchi A (2010). Injury-induced neural stem/progenitor cells in post-stroke human cerebral cortex. Eur J Neurosci.

[7] Kaneko N, Marín O, Koike M, Hirota Y, Uchiyama Y, Wu JY, Lu Q, Tessier-Lavigne M, Alvarez-Buylla A, Okano H, Rubenstein JL, Sawamoto K (2010). New neurons clear the path of astrocytic processes for their rapid migration in the adult brain. Neuron.

[8] García-González D, Clemente D, Coelho M, Esteban PF, Soussi-Yanicostas N, de Castro F (2010). Dynamic roles of FGF-2 and Anosmin-1 in the migration of neuronal precursors from the subventricular zone during pre- and postnatal development. Exp Neurol.

[9] Kojima T, Hirota Y, Ema M, Takahashi S, Miyoshi I, Okano H, Sawamoto K (2010). Subventricular zone-derived neural progenitor cells migrate along a blood vessel scaffold toward the post-stroke striatum. Stem Cells..

[10] Osman AM, Porritt MJ, Nilsson M, Kuhn HG (2011). Long-term stimulation of neural progenitor cell migration after cortical ischemia in mice. Stroke..

[11] Liu XS, Zhang ZG, Zhang RL, Gregg SR, Wang L, Yier T, Chopp M (2007). Chemokine ligand 2 (CCL2) induces migration and differentiation of subventricular zone cells after stroke. J Neurosci Res.

[12] Wang F, Shen Y, Tsuru E, Yamashita T, Baba N, Tsuda M, Maeda N, Sagara Y (2015). Syngeneic transplantation of newborn splenocytes in a murine model of neonatal ischemia-reperfusion brain injury. J Matern Fetal Neonatal Med.

[13] Sallusto F, Baggiolini M (2008). Chemokines and leukocyte traffic. Nat Immunol.

[14] Parent JM, Vexler ZS, Gong C, Derugin N, Ferriero DM (2002). Rat forebrain neurogenesis and striatal neuron replacement after focal stroke. Ann Neurol.

[15] Ohab JJ, Fleming S, Blesch A, Carmichael ST (2006). A neurovascular niche for neurogenesis after stroke. J Neurosci.

[16] Yamashita T, Ninomiya M, Hernández Acosta P, García-Verdugo JM, Sunabori T, Sakaguchi M, Adachi K, Kojima T, Hirota Y, Kawase T, Araki N, Abe K, Okano H, Sawamoto K (2006). Subventricular zone-derived neuroblasts migrate and differentiate into mature neurons in the post-stroke adult striatum. J Neurosci.

[17] Thored P, Arvidsson A, Cacci E, Ahlenius H, Kallur T, Darsalia V, Ekdahl CT, Kokaia Z, Lindvall O (2006). Persistent production of neurons from adult brain stem cells during recovery after stroke. Stem Cells.

[18] Studer L, Csete M, Lee SH, Kabbani N, Walikonis J, Wold B, McKay R (2000). Enhanced proliferation, survival, and dopaminergic differentiation of CNS precursors in lowered oxygen. J Neurosci.

[19] Kalluri HS, Eickstaedt J, Dempsey RJ (2007). Oxygen glucose deprivation inhibits the growth and ERK phosphorylation of neural progenitor cells in vitro. Neurosci Lett.

[20] Cartier L, Hartley O, Dubois-Dauphin M, Krause KH (2005). Chemokine receptors in the central nervous system: role in brain inflammation and neurodegenerative diseases. Brain Res Brain Res Rev.

[21] Tran PB, Ren D, Veldhouse TJ, Miller RJ (2004). Chemokine receptors are expressed widely by embryonic and adult neural progenitor cells. J Neurosci Res.

[22] Ma Q, Jones D, Borghesani PR, Segal RA, Nagasawa T, Kishimoto T, Bronson RT, Springer TA (1998). Impaired B-lymphopoiesis, myelopoiesis, and derailed cerebellar neuron migration in CXCR4- and SDF-1-deficient mice. Proc Natl Acad Sci U S A.

[23] Lu M, Grove EA, Miller RJ (2002). Abnormal development of the hippocampal dentate gyrus in mice lacking the CXCR4 chemokine receptor. Proc Natl Acad Sci U S A.

[24] Robin AM, Zhang ZG, Wang L, Zhang RL, Katakowski M, Zhang L, Wang Y, Zhang C, Chopp M (2006). Stromal cell-derived factor 1alpha mediates neural progenitor cell motility after focal cerebral ischemia. J Cereb Blood Flow Metab.

[25] Peng H, Huang Y, Rose J, Erichsen D, Herek S, Fujii N, Tamamura H, Zheng J (2004). Stromal cell-derived factor 1-mediated CXCR4 signaling in rat and human cortical neural progenitor cells. J Neurosci Res.

[26] Gordon RJ, McGregor AL, Connor B (2009). Chemokines direct neural progenitor cell migration following striatal cell loss. Mol Cell Neurosci.

[27] Lum M, Croze E, Wagner C, McLenachan S, Mitrovic B, Turnley AM (2009). Inhibition of neurosphere proliferation by IFNgamma but not IFNbeta is coupled to neuronal differentiation. J Neuroimmunol.

[28] Im SH, Yu JH, Park ES, Lee JE, Kim HO, Park KI, Kim GW, Park CI, Cho SR (2010). Induction of striatal neurogenesis enhances functional recovery in an adult animal model of neonatal hypoxic-ischemic brain injury. Neuroscience.

[29] Millard CJ, Ludeman JP, Canals M, Bridgford JL, Hinds MG, Clayton DJ, Christopoulos A, Payne RJ, Stone MJ (2014). Structural basis of receptor sulfotyrosine recognition by a CC chemokine: the N-terminal region of CCR3 bound to CCL11/eotaxin-1. Structure.

[30] Fryer AD, Stein LH, Nie Z, Curtis DE, Evans CM, Hodgson ST, Jose PJ, Belmonte KE, Fitch E, Jacoby DB (2006). Neuronal eotaxin and the effects of CCR3 antagonist on airway hyperreactivity and M2 receptor dysfunction. J Clin Invest.

[31] Villeda SA, Luo J, Mosher KI, Zou B, Britschgi M, Bieri G, Stan TM, Fainberg N, Ding Z, Eggel A, Lucin KM, Czirr E, Park JS, Couillard-Després S, Aigner L, Li G, Peskind ER, Kaye JA, Quinn JF, Galasko DR, Xie XS, Rando TA, Wyss-Coray T (2011). The ageing systemic milieu negatively regulates neurogenesis and cognitive function. Nature.

[32] Lezi E, Burns JM, Swerdlow RH (2014). Effect of high-intensity exercise on aged mouse brain mitochondria, neurogenesis, and inflammation. Neurobiol Aging.

[33] de Miranda AS, Brant F, Campos AC, Vieira LB, Rocha NP, Cisalpino D, Binda NS, Rodrigues DH, Ransohoff RM, Machado FS, Rachid MA, Teixeira AL (2015). Evidence for the contribution of adult neurogenesis and hippocampal cell death in experimental cerebral malaria cognitive outcome. Neuroscience..

[34] Parajuli B, Horiuchi H, Mizuno T, Takeuchi H, Suzumura A (2015). CCL11 enhances excitotoxic neuronal death by producing reactive oxygen species in microglia. Glia.

[35] Erickson MA, Morofuji Y, Owen JB, Banks WA (2014). Rapid transport of CCL11 across the blood-brain barrier: regional variation and importance of blood cells. J Pharmacol Exp Ther.

[36] Adzemovic MZ, Öckinger J, Zeitelhofer M, Hochmeister S, Beyeen AD, Paulson A, Gillett A, Thessen Hedreul M, Covacu R, Lassmann H, Olsson T, Jagodic M (2012). Expression of Ccl11 associates with immune response modulation and protection against neuroinflammation in rats. PLoS One.

[37] Kodali RB, Kim WJ, Galaria II, Miller C, Schecter AD, Lira SA, Taubman MB (2004). CCL11 (Eotaxin) induces CCR3-dependent smooth muscle cell migration. Arterioscler Thromb Vasc Biol.

[38] Maysami S, Nguyen D, Zobel F, Heine S, Höpfner M (2006). Oligodendrocyte precursor cells express a functional chemokine receptor CCR3: implications for myelination. Stangel M J Neuroimmunol.

[39] Reynolds R, Dawson M, Papadopoulos D, Polito A, Di Bello IC, Pham-Dinh D, Levine J (2002). The response of NG2-expressing oligodendrocyte progenitors to demyelination in MOG-EAE and MS. J Neurocytol.

[40] Bozzali M, Wrabetz L (2004). Axonal signals and oligodendrocyte differentiation. Neurochem Res.

[41] Ross R (1995). Cell biology of atherosclerosis. Annu Rev Physiol..

[42] Furukawa T, Matsui N, Fujita K, Nodera H, Shimizu F, Miyamoto K, Takahashi Y, Kanda T, Kusunoki S, Izumi Y, Kaji R (2015). CSF cytokine profile distinguishes multifocal motor neuropathy from progressive muscular atrophy. Neurol Neuroimmunol Neuroinflamm.

