# Supplementary figures and images for: CCL11 promotes migration and proliferation of mouse neural progenitor cells
Source: Stem Cell Res Ther. 2017 Feb 7;8:26. doi: 10.1186/s13287-017-0474-9 (PMC5297016; doi:10.1186/s13287-017-0474-9)

Supplementary Figure2

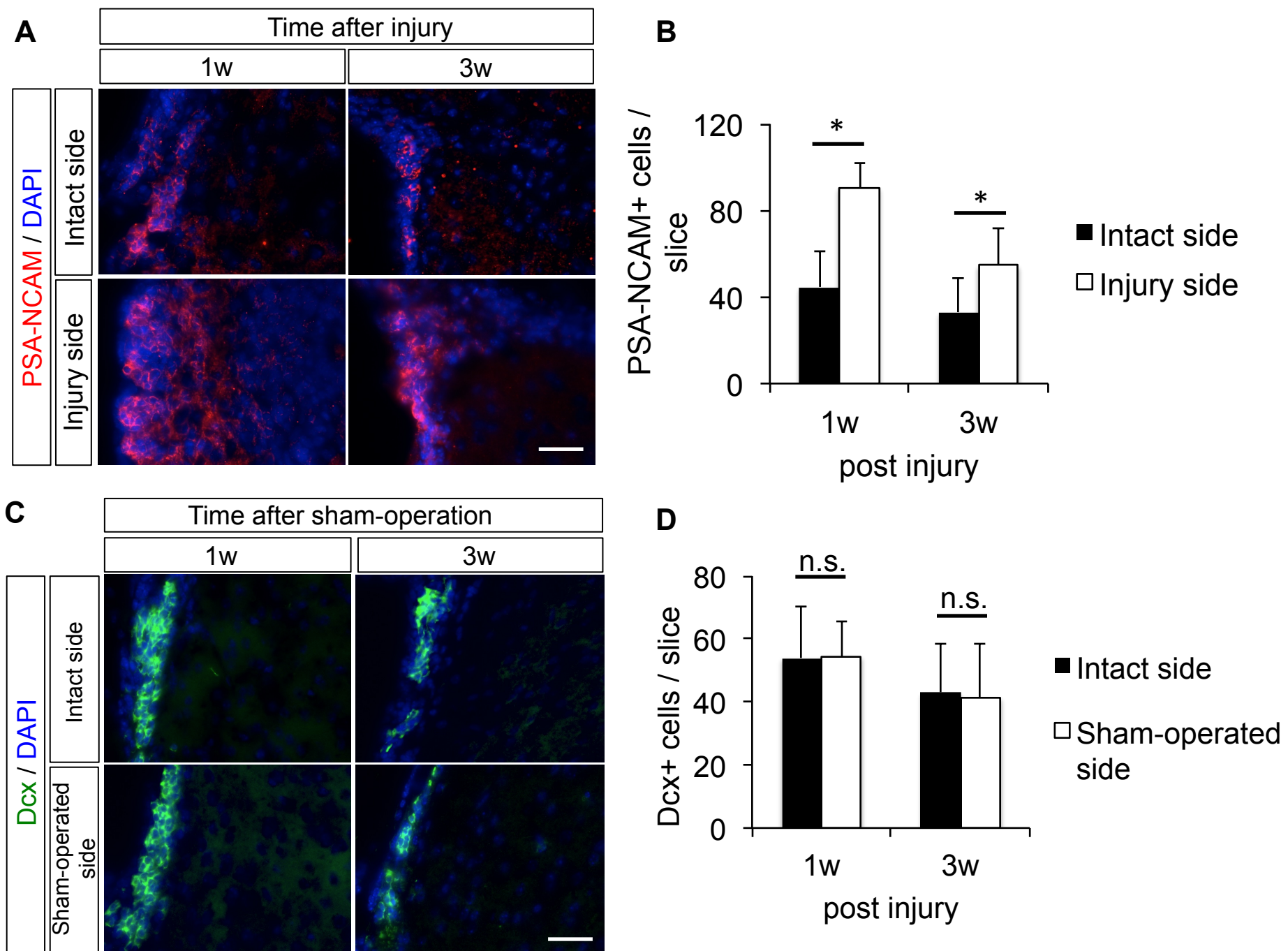

Supplement: Additional file 2: Figure S2. — The immunostaining in the model mice of neonatal hypoxic-ischemic brain injury and the sham-operated mice. (A) PSA-NCAM immunostaining image of SVZ in the model mouse. Red: PSA-NCAM; blue: DAPI. Scale bar = 50 μm. (B) Number of PSA-NCAM-positive cells per slice. N = 8. The data are presented as the mean numbers of PSA-NCAM-positive cells in the injury side and the intact side ± SD. * P < 0.05. (C) Dcx immunostaining image of SVZ in the sham-operated mouse. N = 6. Green: Dcx; blue: DAPI. Scale bar = 50 μm. (D) Number of Dcx-positive cells per slice. N = 8. The data are presented as the mean numbers of Dcx-positive cells in the sham-operated side and the intact side ± SD. n.s. not significant. (PDF 655 kb) [file 13287_2017_474_MOESM2_ESM.pdf]
